# Downregulation of *BmSTAT* transcription factor promoted nucleopolyhedrovirus replication in *Bombyx mori*

**DOI:** 10.3389/fmicb.2024.1485951

**Published:** 2024-10-23

**Authors:** Wenjuan Liang, Li Zhou, Zhuo Dang, Shiyuan Wang, Ping Zhao, Qingyou Xia, Zhongyan Lu

**Affiliations:** ^1^Integrative Science Center of Germplasm Creation in Western China (CHONGQING) Science City, Biological Science Research Center, Southwest University, Chongqing, China; ^2^Chongqing Engineering and Technology Research Center for Novel Silk Materials, Southwest University, Chongqing, China

**Keywords:** BmSTAT, BmNPV, transgene, *Bombyx mori*, immune response

## Abstract

The Janus kinase/signal transducer and activator of transcription (JAK/STAT) signaling pathway plays a crucial role in the viral immune processes of organisms, with STAT being a key transcription factor downstream in this pathway. The *STAT* gene of *Bombyx mori* has two splicing forms, named *BmSTAT-S* and *BmSTAT-L*. This study compared the effects of the two splicing forms on *Bombyx mori* nucleopolyhedrovirus (BmNPV) infection through cell-level interference and further explored whether *BmSTAT* participates in the immune response to BmNPV infection via transgenic intervention at the individual level. Our research results indicated that BmNPV upregulates the expression of *BmSTAT-S* and *BmSTAT-L* in *Bombyx mori* BmE cells and larvae. Furthermore, BmE cells with interference of *BmSTAT-S* or *BmSTAT-L* displayed significantly higher expression levels of the viral gene *GP41* and increased viral fluorescence compared to the control group after 48 h of infection with BmNPV. Then, we constructed transgenic silkworms with genetic interference, and the results showed that both the transgenic silkworms with systemic interference and midgut-specific interference of the two splice forms of *BmSTAT* exhibited significantly reduced survival rates and increased viral replication numbers after infection with BmNPV. The above results indicated that the *BmSTAT* gene is involved in the immune response of *Bombyx mori* to BmNPV and these findings lay the foundation for further research on the mechanism of JAK/STAT signaling pathway involvement in BmNPV infection.

## 1 Introduction

*Bombyx mori*, a significant economic insect, holds considerable importance in sericulture and life science research (Goldsmith et al., 2005; Jiang et al., 2021b). In China, diseases in silkworms account for over 20% of the annual cocoon yield losses, with viral infections contributing to more than 80% of these losses, predominantly due to the *Bombyx mori* nucleopolyhedrovirus (BmNPV) induced hemorrhagic septicemia (Jiang and Xia, 2014; Jiang, 2021). BmNPV, a rod-shaped virus with circular double-stranded DNA, occurs across various silkworm-producing regions globally, particularly causing outbreaks during the summer and autumn seasons that lead to significant production losses. Although studies have indicated that multiple signaling pathways, including RNA interference (RNAi), the stimulator of interferon genes (STING) signaling pathway, and the NF-kB signaling pathway, are involved in the immune response of *Bombyx mori* to viral infections (Jiang, 2021), the immune mechanisms between *Bombyx mori* and BmNPV have not yet been effectively elucidated.

The Janus kinase/signal transducer and activator of transcription (JAK/STAT) signaling pathway is a ubiquitously expressed intracellular signal transduction pathway, with related proteins and fundamental functions are relatively conserved across organisms. This pathway has been demonstrated to participate in numerous critical biological processes including cell proliferation, differentiation, apoptosis, and immune regulation (Hombría and Brown, 2002; Myllymäki and Rämet, 2014; Herrera and Bach, 2019). The human STAT protein family consists of seven members, and comparative studies on the sequences of different *STAT* gene products reveal their common origin and evolutionary process. However, research discovered that these seven *STAT* exhibited a wide range of biological functions. Specifically, mice with *STAT1* or *STAT2* genes knocked out exhibit impaired antiviral responses, while the knockout of *STAT3* results in embryonic lethality. Furthermore, the deletion of *STAT4* leads to impaired T-helper 1 (Th1) cells development, and the absence of *STAT5* causes defects in mammary gland development, infertility, and the absence of natural killer (NK) cells. Additionally, the knockout of *STAT6* impairs T-helper 2 (Th2) cells development (Ihle, 1996; Morris et al., 2018). In *Anopheles gambiae*, two *STAT* genes exist: *AgSTAT-A* and a second intronless *STAT* gene, *AgSTAT-B*, which is produced by replication of *AgSTAT-A in vivo*. *AgSTAT-B* acts upstream to regulate the expression of *AgSTAT-A*, jointly responding to bacterial and *Plasmodium* infections (Gupta et al., 2009). In *Caenorhabditis elegans*, STA-1 regulates TGF-β signaling, while STA-2 plays a crucial role in maintaining the integrity and barrier function of the epidermis (Wang and Levy, 2006; Zhang et al., 2015). Additionally, in the fall armyworm *Spodoptera frugiperda*, two splice variants of *STAT* are found in the Sf9 cell line derived from pupal ovarian tissues, but no studies have yet demonstrated functional differences between these splice variants (Yan et al., 1996; Yeh et al., 2008). In *Drosophila*, *STAT* is critically important for various developmental processes, including embryonic segmentation (Hou et al., 1996), formation of wings and eyes (Hou et al., 1996), development of the hindgut and midgut (Schindler and Darnell, 1995), proliferation and differentiation of blood cells (Myllymäki and Rämet, 2014), and maintenance of germline stem cells (Hombría and Brown, 2002).

In addition, the JAK/STAT pathway has also been proven to be widely involved in the viral immune response of various organisms. In mammals, the JAK/STAT pathway can induce the upregulation of multiple interferon-stimulated genes (ISGs), thereby rapidly eliminating viruses within infected cells (Iwasaki and Pillai, 2014; Schoggins et al., 2014). Moreover, *STAT* plays a pivotal role in the antiviral defense of *Drosophila*, studies found that the number of factors specifically binding to the optimal DNA binding site of *STAT92E* in whole fly nuclear extracts of *Drosophila* infected with *Drosophila* C virus (DCV) increased, indicated that the infection of *Drosophila* with DCV induced the DNA binding activity of *STAT* (Yan et al., 1996; Dostert et al., 2005; Kemp et al., 2013). The infection with invertebrate iridescent virus 6 (IIV-6) also triggers the JAK/STAT pathway, which is crucial for the host’s defense against IIV-6 infection (West and Silverman, 2018). The pathway has also been shown to contribute to the immune response between *Aedes aegypti* and dengue virus (DENV)(Souza-Neto et al., 2009), and *Culex* mosquitoes and West Nile virus (WNV) (Paradkar et al., 2012). Additionally, studies indicated that the JAK/STAT pathway can promote viral proliferation during viral infection responses. For instance, the cytokine receptor Domeless in *Litopenaeus vannamei* (*LvDOME*) may be hijacked by the white spot syndrome virus (WSSV), facilitating viral replication (Yan et al., 2015), the *CqDOME* in *Cherax quadricarinatus* plays a crucial role in enhancing WSSV infection (Liu et al., 2020), and WSSV utilizes the transcription factor *PmSTAT* in *Penaeus monodon* to augment the expression of viral genes within host cells (Liu et al., 2007). These observations suggest that the immunological functions and mechanisms of the JAK/STAT pathway vary among different species.

The fundamental components of the JAK/STAT signaling pathway in *Bombyx mori* have been identified, including key genes such as *BmDOME*, *BmSTAT*, and *BmHOP*. The *BmSTAT* gene in *Bombyx mori* exists in two splice forms, named *BmSTAT*-*S* and *BmSTAT*-*L*. Previous studies reported that the open reading frames (ORFs) of *BmSTAT-S* and *BmSTAT-L* comprise 2,202 and 2,313 nucleotides, encoding 733 and 770 amino acids, respectively. The last 44 amino acid residues of *BmSTAT-L* differ from the final 7 amino acid residues of *BmSTAT-S*, with *BmSTAT-S* being spliced from exons 1–19 and *BmSTAT-L* from exons 1–18 and exon 20. Amino acid homology analysis revealed that the *BmSTAT* gene shares similarities of 92, 43, 35, and 42% with *Spodoptera frugiperda STAT* (*SfSTAT*), *Danio rerio STAT* (*DrSTAT*), *Drosophila melanogaster STAT* (*DmSTAT*), and *Homo sapiens STAT* (*HsSTAT*), respectively (Zhang et al., 2016). Research indicated that JAK/STAT signaling pathway participates in cellular proliferation (Hu et al., 2015), and the development of wing primordia in *Bombyx mori* (Zhang et al., 2020). Moreover, it had been demonstrated to play a significant role in the immune response of *Bombyx mori*, BmNPV infection can activate the JAK/STAT pathway (Liu et al., 2015), and under *Beauveria bassiana* stress, this pathway may be involved in the synthesis and secretion of antifungal substances (Geng et al., 2016; Zhang et al., 2016). Inhibition of heat shock protein 90 (Hsp90) can suppress BmNPV proliferation in *Bombyx mori* and leads to the upregulation of *STAT* and downregulation of suppressor of cytokine signalling protein 2 (*SOCS2*) and *SOCS6*, suggesting a potential cooperative role of Hsp90 with the JAK/STAT pathway in viral resistance (Shang et al., 2020). However, whether the key downstream transcription factor *BmSTAT* in the JAK/STAT signaling pathway participates in the BmNPV infection and its mechanisms has not yet been reported. This study explored the relationship between *BmSTAT* and BmNPV replication from the cellular and individual levels, laying a foundation for further research on the mechanism of JAK/STAT pathway involvement in BmNPV infection and how to inhibit the replication of BmNPV in *Bombyx mori*.

## 2 Materials and methods

### 2.1 Cell, silkworm strain, and viruses

The silkworm embryonic cell line BmE, the silkworm strain Dazao (DZ), wild BmNPV, and BmNPV-GFP expressing green fluorescent protein were maintained at Biological Science Research Center (Southwest University, Chongqing, China). BmE cells were cultured at 27°C in Grace medium. Silkworms were fed with mulberry leaves under conditions of 25°C. The silkworm larvae were orally inoculated with wild BmNPV and BmE cells were infected with BmNPV-GFP.

### 2.2 Subcellular localization

The cDNA of silkworm gonad was used as the template for polymerase chain reaction (PCR), and the cell expression vectors pSL1180 [Hr3-A4-RED-*BmSTAT-S*-SV40] and pSL1180 [Hr3-A4-RED-*BmSTAT-L*-SV40] were constructed. The related sequences are shown in Supplementary data and the basic vector pSL1180 [Hr3-A4-RED-SV40] is conserved in our laboratory. Cell transfection was conducted using X-tremeGENE HP DNA transfection reagent (Roche, Mannheim, Germany) according to the manufacturer’s instructions, and incubated for 8 h with serum-free media without antibiotics. Then, cells were cultured for another 48 h in fresh completed medium before other treatments. After 48 h of transfection, the cells were fixed for 10 min with 4% paraformaldehyde at room temperature and washed three times with phosphate buffered saline (PBS). Then cells were incubated with 4′-6-diamidino-2-phenylindole (DAPI) for 10 min, and observed under a fluorescence microscope after washing three times with PBS (Kang et al., 2021).

### 2.3 Viral infection

Monolayer cultures of BmE cells were seeded in 12-well plates for 12 h. BmNPV-GFP was added to the cells at a multiplicity of infection (MOI) of 1 (200 μl/well in 12-well plates) and the supernatant was removed after 1 h of absorption. Then, fresh complete medium was added, and culturing continued at 27°C (Wang et al., 2017). Cell RNA was extracted at 0, 3, 6, 12, and 24 h after BmNPV infection, and quantitative polymerase chain reaction (qPCR) was performed to detect the expression levels of *BmSTAT-S* and *BmSTAT-L*. For transfected cells, infected with BmNPV-GFP at 48 h after transfection, the operation method is the same as above. Total DNA was extracted for qPCR analysis of BmNPV gene *GP41* and control gene glyceraldehyde3-phosphate dehydrogenase (*BmGAPDH*) (Guo et al., 2016) at 24 and 48 h post infection (hpi). The viral fluorescence was observed at 72 hpi.

The transgenic and wild silkworms were raised to fourth instar, 300 healthy larvae were randomly selected from each transgenic strain and non-transgenic strain. The experimental group was infected with BmNPV, and the control group was not infected with the virus. Each treatment contained three replicates, and each replicate contained 50 larvae. Orally infected with wild BmNPV using 2 × 10^6^ occlusion bodies (OB)/larva. Total DNA was extracted from six larvae of transgenic and non-transgenic silkworms at 48 h after infection with BmNPV, which was used for qPCR analysis of the BmNPV gene *GP41* and the control gene *BmGAPDH*. The mortality of larvae was recorded daily until day 10 post infection (Guo et al., 2019).

### 2.4 Quantitative polymerase chain reaction analysis

The total RNA was extracted using Trizol (Invitrogen, USA). 1 μg of total RNA was used for cDNA synthesis following the manufacturer’s protocol of the PrimeScript RT reagent Kit with gDNA Eraser (Perfect Real Time; TaKaRa, Japan). RNA samples were reverse transcribed into cDNA and used as templates. Genomic DNA samples were diluted with ddH_2_O to 200 ng/μl and used as templates. qPCR was performed with a NovoStart SYBR qPCR SuperMix Plus (Novoprotein, China) and a 7500 Fast Real-time PCR System (Applied Biosystems, USA). The silkworm *BmGAPDH* gene (Guo et al., 2019) was used as the internal control. All primer sequences are shown in Supplementary Table S1. All experiments were independently performed with three biological replicates, and each detection was performed three times. Finally, relative gene expression levels were analyzed using the 2^−△△Ct^ method, and statistical analyses were performed using GraphPad Prism version 8 (Tang et al., 2020).

### 2.5 RNA interference

For RNA interference (RNAi) of *BmSTAT*, about 500 bp fragment was amplified by PCR from cDNA using a pair of primers with T7 RNA polymerase-binding site attached to the 5′-end of each primer for *BmSTAT* (related primers are shown in Supplementary Table S1). First, the DNA fragment was amplified using PCR, and the double-stranded RNAs (dsRNAs) were synthesized *in vitro* at 37°C by T7 RNA polymerase (Promega, Madison, WI, USA) for 12 h using the DNA fragment as a template (Kang et al., 2021). The Red gene dsRNA (dsRed) was used as a control. Specific sequence for *BmSTAT-S* and *BmSTAT-L* were selected, and small interfering RNAs (siRNAs) were designed and synthesized by Sangon Biotech (Shanghai, China). Related sequences are shown in Supplementary Table S2. A 2 μg sample of dsRNA or siRNA was transfected into BmE cells. After 48 h, RNA was extracted for qPCR analysis.

### 2.6 Establishment and detection of transgenic interference silkworm strains

Transgenic interference vectors were constructed using dsRNA fragments. The synthesis of pMV [*Bam*HI-ds*BmSTAT* + A3intron-*Eco*R I] and pMV [*Eco*R I-FXHBds*BmSTAT*-*Not* I] was carried out at Sangon Biotech (Shanghai, China). ds*BmSTAT* and FXHBds*BmSTAT* are reverse complementary sequences. Then, the two target fragments were connected to vectors for whole-body overexpress, pSL1180 [Hr3-A4-SV40], and midgut specific overexpression, pSL1180 [P3P + 5UI-SV40], producing two expression vectors, pSL1180 [Hr3-A4-ds*BmSTAT*-SV40] and pSL1180 [P3P + 5UI-ds*BmSTAT*-SV40]. Finally, the fragments were connected to the piggyBac [3 × P3-EGFP] vector, generating the piggyBac [3 × P3-EGFP, A4-ds*BmSTAT*] systemic transgenic interference vector and the piggyBac [3 × P3-EGFP, P3P + 5UI-ds*BmSTAT*] midgut-specific transgenic interference vector. The A3 intron was cloned from the genomic DNA of DZ silkworm and used as a spacer between antisense and sense fragments. The related sequences are shown in the Supplementary data and the basic vectors are conserved in our laboratory.

Non-diapause DZ embryos were used for microinjection with the transgenic vector, this generation was called G0. The G1 embryos were screened for GFP-positive expression using a fluorescent microscope (Olympus, Japan). Two transgenic lines were obtained and named as A4-ds*BmSTAT* and Mg-ds*BmSTAT*. RNA from the midgut was extracted from A4-ds*BmSTAT*, Mg-ds*BmSTAT* and non-transgenic silkworm on the third day of fifth instar for qPCR analysis of *BmSTAT-S*, *BmSTAT-L* and the control *BmGAPDH*. Each detection was performed three times. All the primers are shown in Supplementary Table S1.

### 2.7 Statistical analysis

Statistical data were presented as means ± standard deviation (SD). Three independent experiments were performed to ensure validity, with at least three samples per test taken for statistical analysis. Statistical analyses were conducted using GraphPad Prism 8.0 (GraphPad Software, La Jolla, CA, USA). Significance (*p* value) was determined using the Student’s *t*-test and denoted as follows: *p < 0.05, **p < 0.01, and ***p < 0.001.

## 3 Results

### 3.1 Expression and localization of *BmSTAT-S* and *BmSTAT-L* in *Bombyx mori*

**Figure 1:**
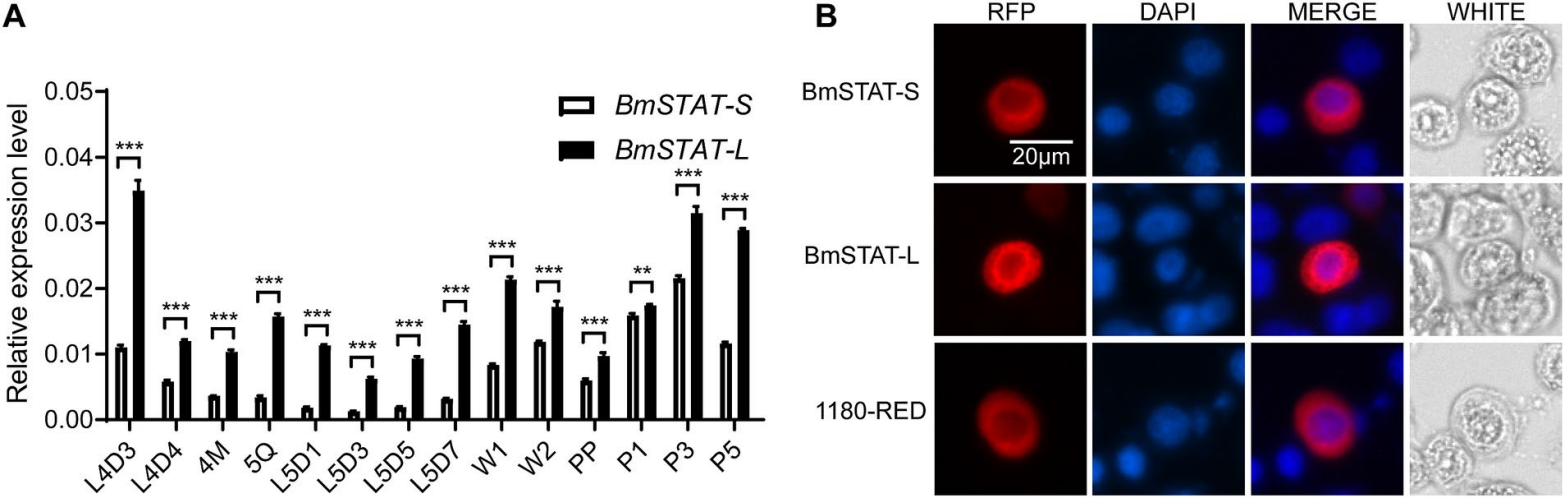
Expression and localization of *BmSTAT-S* and *BmSTAT-L* in *B. mori*. **(A)** Expression profile analysis of *BmSTAT* in midgut. L4D3 and L4D4 represent day 3 and day 4 of the fourth instar; 4 M: the fourth molting; 5Q: fifth instar larva; L5D1, L5D3, L5D5, and L5D7 correspond to day 1, day 3, day 5, and day 7 of the fifth instar, respectively; W1 and W2 represent day 1 and day 2 at the wandering stage; PP: prepupa; P1, P3, P5 represent day 1, day 3, day 5 of the pupal stage, respectively. **(B)** Subcellular localization analysis of BmSTAT. RFP: Red fluorescence protein fusion expression vector; DAPI: Stained nucleus (blue); 1,180-RED is the control group. Data are presented as means ± SD (*n* = 3). Significance levels: *p < 0.05, **p < 0.01, and ***p < 0.001; ns indicates no significance.

The midgut is an important organ of innate immunity in silkworms, and to resist the infection of pathogenic microorganisms, the silkworm midgut has developed a complete immune response system. In our earlier study, we found that the expression level of *BmSTAT* in the midgut was relatively high (Wang et al., 2024), suggesting that it might be involved in the immune response to pathogens. To explore the temporal expression patterns of *BmSTAT* in the midgut of *Bombyx mori*, quantitative reverse transcription PCR (qRT-PCR) was employed to monitor the midgut from day-3 of the fourth instar to day-5 of the pupal stage. The findings revealed that both splice forms of *BmSTAT* were expressed at all stages examined, with *BmSTAT-L* consistently showing higher expression levels than *BmSTAT-S*. The expression levels of both splice forms in the midgut began to decrease from day-3 of the fourth instar, followed by a gradual increase by day-3 of the fifth instar (Figure 1A).

To investigate the subcellular localization of BmSTAT, the *BmSTAT* gene was transfected into *Bombyx mori* embryonic cells (BmE). The results revealed that both splice variants of BmSTAT predominantly localize in the cytoplasm of BmE cells, with minor distribution also observed in the nucleus (Figure 1B).

### 3.2 The expression levels of *BmSTAT-S* and *BmSTAT-L* are upregulated by BmNPV induction in *Bombyx mori*

**Figure 2:**
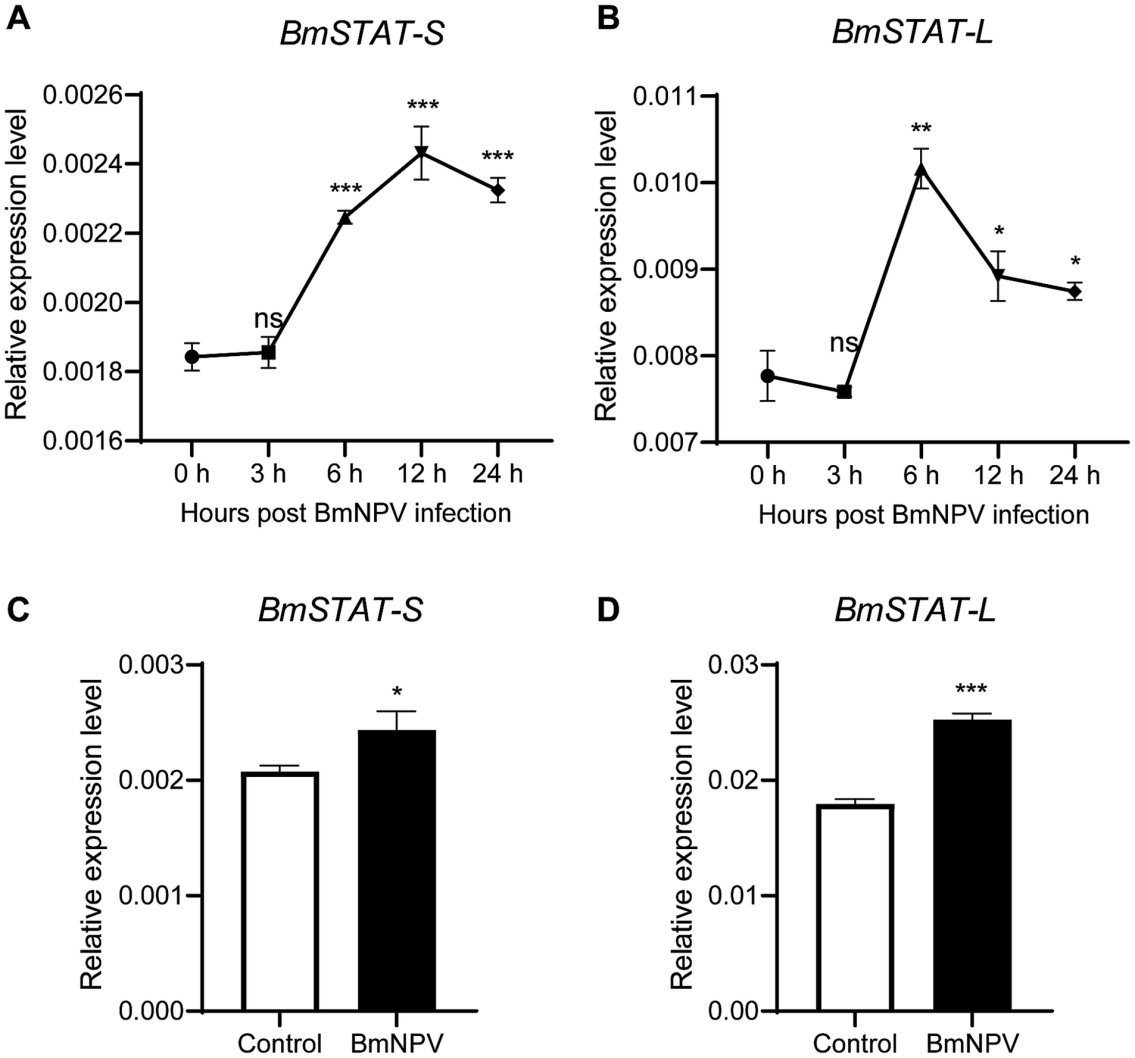
qRT-PCR analysis of *BmSTAT-S* and *BmSTAT-L* after BmNPV infection. **(A,B)** Detection of *BmSTAT-S* and *BmSTAT-L* in cells post-BmNPV infection. BmE cells were infected with BmNPV-GFP, and RNA was extracted at 0, 3, 6, 12, and 24 hpi to detect the expression of *BmSTAT-S* and *BmSTAT-L*. **(C,D)** Detection of *BmSTAT-S* and *BmSTAT-L* in silkworm post-BmNPV infection. Fourth-instar,1-day-old silkworm larvae were orally infected with wild-type BmNPV, and RNA was extracted at 48 hpi to detect *BmSTAT-S* and *BmSTAT-L* expression. Control, non-infected silkworms; BmNPV, infected silkworms. Data are presented as means ± SD (*n* = 3). Significance levels: *p < 0.05, **p < 0.01, and ***p < 0.001; ns indicates no significance.

To determine whether *BmSTAT* responds to BmNPV infection, BmE cells were infected with BmNPV-GFP (MOI = 1), and fourth-instar larvae were infected with wild-type BmNPV (2 × 10^6^ OB/larva). The transcriptional levels of *BmSTAT-S* and *BmSTAT-L* were assessed using qRT-PCR. The results showed that in BmE cells, the expression levels of *BmSTAT-S* and *BmSTAT-L* significantly increased at 6, 12, and 24 h post-infection (Figures 2A,B). In *Bombyx mori* larvae, the expression levels of *BmSTAT-S* and *BmSTAT-L* were significantly elevated 48 h post-infection, reaching 1.18 and 1.41 times the control levels, respectively (Figures 2C,D). These experimental results indicate that BmNPV can activate the JAK/STAT pathway and induce an increase in the expression of *BmSTAT-S* and *BmSTAT-L*.

### 3.3 Interference of *BmSTAT-S*, *BmSTAT-L* promotes BmNPV proliferation in BmE cells

**Figure 3:**
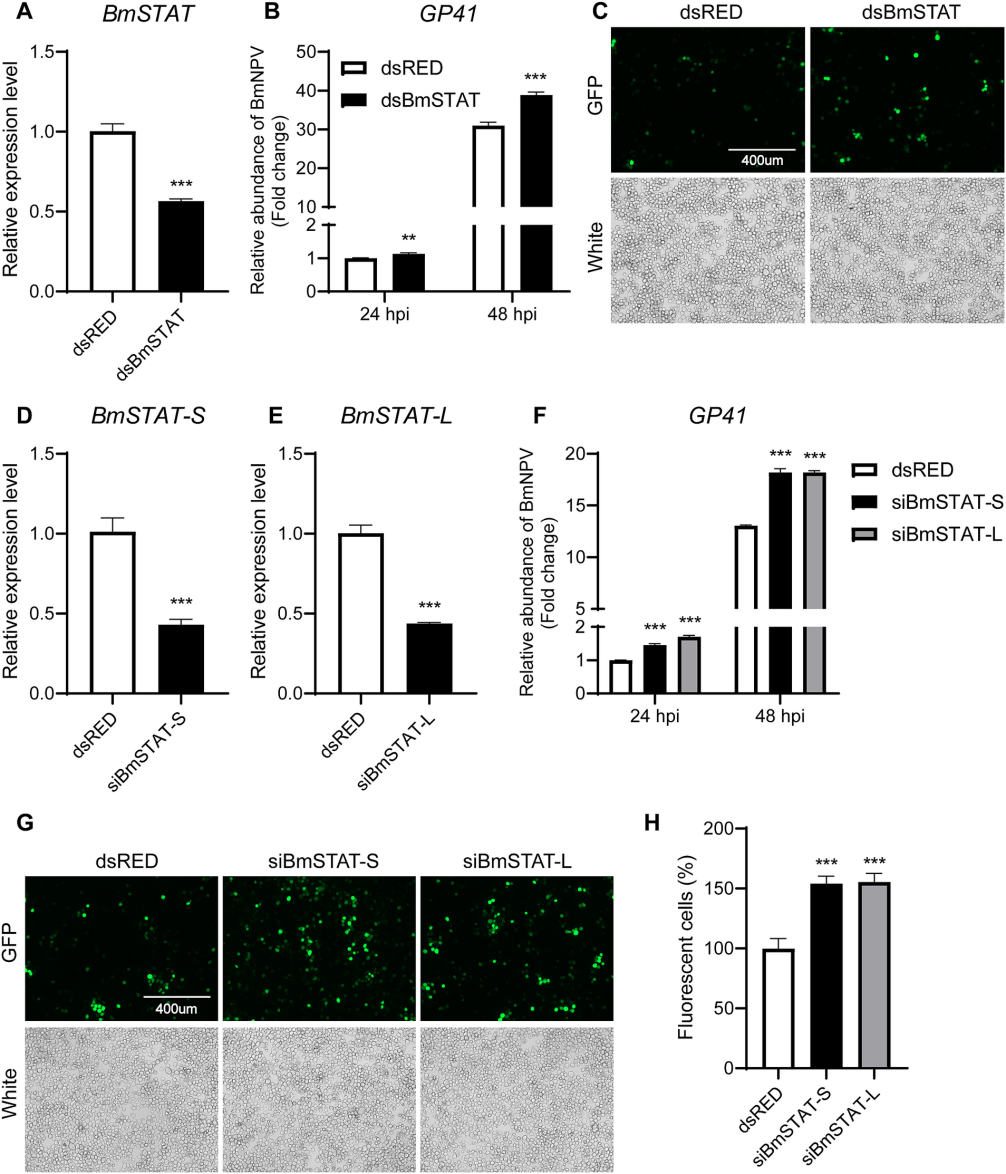
The downregulation of *BmSTAT-S* or *BmSTAT-L* promoted BmNPV multiplication. **(A)** qRT-PCR analysis of *BmSTAT* expression in BmE cells after transfection with ds*BmSTAT* and dsRED. **(B)** Analysis of viral DNA content. The cells were infected with BmNPV-GFP (MOI = 1) 48 h after transfection with dsRed and ds*BmSTAT*. Total DNA was extracted from infected cells at 24 and 48 hpi. The cumulative viral DNA content was assessed by qPCR analysis of *GP41*. **(C)** Observation of viral fluorescence. The green fluorescence of the virus was observed at 72 h after infection. **(D)** Analysis of *BmSTAT-S* expression by qRT-PCR after interference with *BmSTAT-S* in BmE cells. **(E)** Analysis of *BmSTAT-L* expression by qPCR after interference with *BmSTAT-L* in BmE cells. **(F)** Analysis of viral DNA content. After interference with *BmSTAT-S* and *BmSTAT-L*, the cells were infected with BmNPV-GFP (MOI = 1), and total DNA was extracted at 24 and 48 h post-infection for qPCR to detect the virus gene *GP41* to determine the proliferation of the virus. **(G)** The green fluorescence of the virus was observed at 72 h after infection. **(H)** Quantification of fluorescent cells. The numbers of infected BmE cells in images shown in **(G)** were counted. The average level of dsRed was set at 100%. The data presented are the means ± SD (*n* = 3). For the significance analysis: **p < 0.01, ***p < 0.001.

To confirm the impact of *BmSTAT* on BmNPV, double-stranded RNA targeting the common sequence of *BmSTAT-S* and *BmSTAT-L* was synthesized. The results demonstrated that, 48 h post-transfection, the transcription level of *BmSTAT* was successfully reduced by 45% (Figure 3A). Cells with successful *BmSTAT* interference were infected with BmNPV (MOI = 1). Compared to the control group transfected with dsRED double-stranded RNA, the experimental group transfected with ds*BmSTAT* showed an increase in viral copy gene *GP41* to 1.13 times and 1.26 times that of the control at 24 and 48 h, respectively (Figure 3B). At 72 h, the number of virus-infected cells exhibiting green fluorescence was significantly higher than that in the control group (Figure 3C).

To investigate the differential effects of the two splice forms of *BmSTAT* on BmNPV, siRNA was employed to specifically target *BmSTAT-S* and *BmSTAT-L* in *Bombyx mori* cells, followed by infection with BmNPV to assess viral proliferation. The siRNAs synthesized reduced the transcription levels of *BmSTAT-S* by 55% and *BmSTAT-L* by 56% (Figures 3D,E). Cells with successful interference of *BmSTAT-S* or *BmSTAT-L* were infected with BmNPV at an MOI of 1. Compared to the control group transfected with dsRED double-stranded RNA, the group with *BmSTAT-S* interference exhibited 1.45 times and 1.40 times the viral copy number at 24 and 48 h, respectively (Figure 3F). For the *BmSTAT-L* interference group, the viral copy numbers were 1.70 times and 1.40 times that of the control at 24 and 48 h, respectively (Figure 3F). Additionally, the number of cells displaying viral green fluorescence significantly increased by 72 h in both the *BmSTAT-S* and *BmSTAT-L* interference groups compared to the control (Figures 3G,H). The experimental results indicated that the interference with either *BmSTAT-S* or *BmSTAT-L* individually, or with both splice forms of *BmSTAT* simultaneously, leads to the enhancement of BmNPV replication in BmE cells.

### 3.4 Transgenic silkworm with *BmSTAT* interference has lower resistance to BmNPV

**Figure 4:**
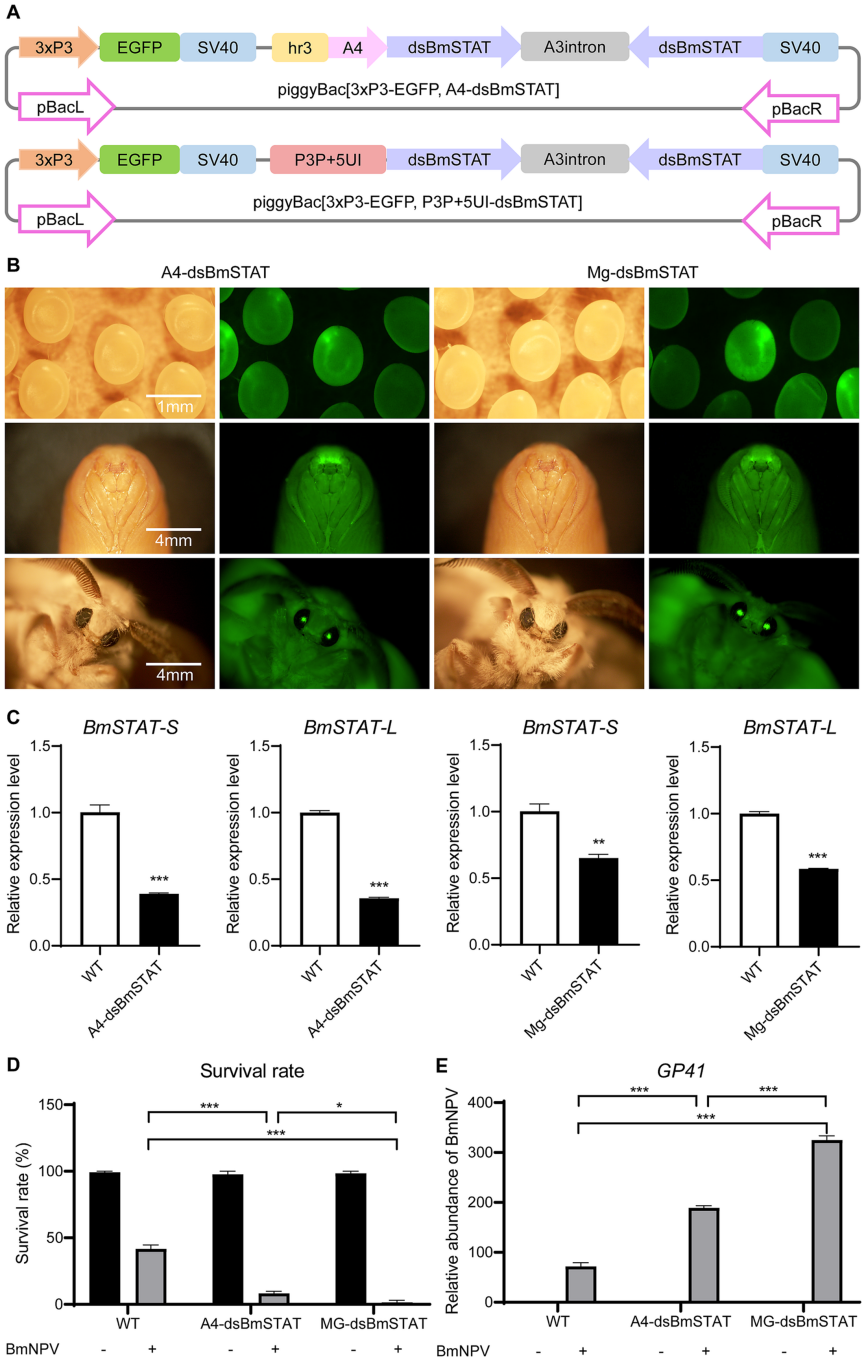
Transgenic silkworms with *BmSTAT* interference had lower resistance to BmNPV. **(A)** Schematic of the piggyBac[3xP3-EGFP, A4-ds*BmSTAT*] and piggyBac [3xP3-EGFP, P3P + 5UI-ds*BmSTAT*] vectors. Hr3, enhancer; A4, systemic promoter; ds*BmSTAT*, double-stranded RNA of *BmSTAT*; A3 intron, a spacer; SV40, polyadenylation signal as terminator; P3P + 5UI, midgut-specific promoter. **(B)** The transgenic silkworm A4-ds*BmSTAT* and Mg-ds*BmSTAT* were observed by fluorescence microscope and photographed. **(C)** RNA was extracted from the midgut of A4-ds*BmSTAT* and Mg-ds*BmSTAT* on the 3rd day of 5th instar, and qRT-PCR was used to detect the expression of *BmSTAT-S* and *BmSTAT-L*. **(D)** The survival rates of A4-ds*BmSTAT* and Mg-ds*BmSTAT* infected with BmNPV. **(E)** Detection of viral replication. After A4-ds*BmSTAT* and Mg-ds*BmSTAT* were infected with BmNPV, the number of viral replications was detected at 48 h. The data presented are the means ± SD (*n* = 3). For the significance analysis: **p < 0.01, ***p < 0.001.

To determine the impact of interfering with *BmSTAT* on BmNPV in individuals, double-stranded RNA was designed to target a common sequence fragment of *BmSTAT-S* and *BmSTAT-L*, and transgenic interference vectors were constructed to obtain transgenic silkworms with gene interference. Initially, we acquired a systemic interference vector, piggyBac [3 × P3-EGFP, A4-ds*BmSTAT*], and a midgut-specific interference vector, piggyBac [3 × P3-EGFP, P3P + 5UI-ds*BmSTAT*] (Figure 4A). Subsequently, both plasmids were microinjected to produce two transgenic interference silkworms, named A4-ds*BmSTAT* (systemic transgenic interference silkworm) and Mg-ds*BmSTAT* (midgut-specific transgenic interference silkworm; Figure 4B). On day three of the fifth instar, midguts were sampled from three randomly selected transgenic positive individuals and wild-type silkworms to extract RNA for qRT-PCR analysis. The expression levels of *BmSTAT-S* and *BmSTAT-L* were found to be significantly reduced in both transgenic lines (Figure 4C).

Fourth instar, day-one larvae of A4-ds*BmSTAT* and Mg-ds*BmSTAT* were fed a BmNPV-infected diet containing 2 × 10^6^ occlusion bodies (OB) per larva. The results demonstrated that both A4-ds*BmSTAT* and Mg-ds*BmSTAT* exhibited reduced survival rates compared to the control group. The survival rate of the control group was 40%, while it was 8% for A4-ds*BmSTAT* and 1% for Mg-ds*BmSTAT* (Figure 4D). Further examination of viral replication 48 h post-infection revealed that the viral copy numbers in both types of transgenic interfered silkworms were significantly higher than those in the control group, being 2.6 and 4.4 times higher, respectively (Figure 4E). These findings indicated that transgenic interference of *BmSTAT* in *Bombyx mori* results in decreased resistance to BmNPV.

## 4 Discussion

The JAK/STAT signaling pathway is one of the most crucial cytokine-mediated signal transduction pathways in organisms and has been demonstrated to participate in numerous key biological processes. Our findings indicate that BmNPV can activate the JAK/STAT signaling pathway and induce an increase in the expression levels of *BmSTAT-S* and *BmSTAT-L*. Moreover, reducing the expression level of *BmSTAT* promotes the proliferation of BmNPV and leads to a decrease in the resistance of silkworms to BmNPV. This demonstrats that *BmSTAT* plays a crucial role in the defense mechanism of silkworms against BmNPV infection and virus replication.

Previously, we discovered that both *BmSTAT-S* and *BmSTAT-L* are highly expressed in the midgut, a crucial immune organ in *Bombyx mori* (Wang et al., 2024). During BmNPV infection, the midgut serves as the initial site of infection and the first line of defense, implying that the *BmSTAT* gene may play a significant role in immune function. Subcellular localization studies showed that *BmSTAT-S* and *BmSTAT-L* were distributed in both the cytoplasm and the nucleus (Figure 1B), consistent with the function of *BmSTAT* as a transcription factor that is activated in the cytoplasm and then enters the nucleus to regulate the expression of downstream target genes. Previous studies have shown that the expression levels of *BmSTAT* in *Bombyx mori* hemolymph significantly increase following infection with both BmNPV and BmBDV (Liu et al., 2015). Post-infection with BmNPV, immunofluorescence of hemocytes showed an increased expression of *BmSTAT-S* (Zhang et al., 2016). We found that after BmNPV infected BmE cells, the expression levels of *BmSTAT-S* and *BmSTAT-L* increased at 6, 12, and 24 h. After stage 4 larvae infected with BmNPV, the expression levels of *BmSTAT-S* and *BmSTAT-L* significantly rose at 48 h post-infection (Figure 2). Understanding the impact of BmNPV on *BmSTAT* facilitates further exploration of the functional relationship between *BmSTAT* and BmNPV. Our research findings also indicated that the simultaneous interference of the two splicing forms of *BmSTAT* in BmE cells promotes the replication of BmNPV. It was further discovered that separate interference of *BmSTAT-S* or *BmSTAT-L* also enhances the proliferation of BmNPV (Figure 3). Therefore, both simultaneous and separate interference yield similar experimental results, suggesting that *BmSTAT* is involved in the *in vivo* response after BmNPV infection and plays a crucial role in resisting BmNPV infection.

Previous studies have shown that the silencing of genes associated with the JAK/STAT signaling pathway leads to a significant increase in viral titers and mortality following viral infections. The knockout of *STAT1* increases susceptibility to mouse hepatitis virus (MHV) and results in a faster mortality rate following vesicular stomatitis virus (VSV) infection compared to controls (Durbin et al., 1996; Park et al., 2000). Knocking out *STAT92E* in *Drosophila* significantly increased lethality after infection with the IIV-6 (West and Silverman, 2018). In addition, studies have shown that the JAK/STAT signaling pathway plays a role in promoting viral proliferation in response to viral infection. The promoter of the shrimp WSSV *ie1* gene contains a *STAT* binding site, and overexpression of *LvDOME* can increase the activity of the *ie1* gene promoter by 20.2 times (Yan et al., 2015). Furthermore, WSSV uses *PmSTAT* as a transcription factor to enhance the expression of viral genes in host cells (Liu et al., 2007). Based on results obtained from the silkworm cellular levels, we chosen to concurrently interfere with both splice forms of *STAT* at the individual level for further experimentation. Our results indicated that reducing *BmSTAT* expression at the individual level significantly decreases survival rates following BmNPV infection and results in copy numbers significantly higher than those in the control group (Figure 4). This suggests that *BmSTAT* may play a role in resisting viral infection during the process of BmNPV infection, similar to that in mammals and *Drosophila*. Additionally, our research found that *Bombyx mori* with midgut-specific interference of the *BmSTAT* gene exhibited a higher mortality rate post-BmNPV infection compared to those with systemic interference. The midgut serves as the initial target organ for BmNPV invasion, where intact occlusion-derived viruses (ODVs) traverse the peritrophic membrane, and nucleocapsids enter the midgut epithelial cells through envelope-mediated membrane fusion, causing primary infection (Jiang et al., 2021a). It is hypothesized that midgut-specific interference of the *BmSTAT* gene facilitates viral invasion, leading to a rapid increase in viral load within the body and consequently higher mortality rates.

In summary, we found that BmNPV upregulates the expression of *BmSTAT-S* and *BmSTAT-L* in both BmE cells and larvae. Interfering with either *BmSTAT-S* or *BmSTAT-L* in BmE cells enhances the proliferation of BmNPV, and systemic and midgut-specific interference of the two splice forms of *BmSTAT* in transgenic *Bombyx mori* results in reduced resistance to BmNPV. Consistent experimental outcomes were obtained at both the cellular and individual levels, indicating that the *BmSTAT* gene is involved in the immune response of *Bombyx mori* to BmNPV and plays a significant role during the immune process. However, the interaction mechanism between the JAK/STAT signaling pathway and BmNPV still requires further study.

## Data Availability

The datasets presented in this study can be found in online repositories. The names of the repository/repositories and accession number (s) can be found in the article/Supplementary material.

## Glossary

*B. mori*: *Bombyx mori*
DZ: Dazao
*BmSTAT*: Signal transducer and activator of transcription
*BmHOP*: Hopscotch
*BmSOCS*: Suppressor of cytokine signaling
*BmPIAS*: Protein inhibitor of activated STAT
*BmDOME*: Cytokine receptor/domeless
BmNPV: *Bombyx mori* nucleopolyhedrovirus
BmNPV-GFP: BmNPV expressing green fluorescent protein
hpi: Hour post infection
OB: Occlusion bodies
PCR: Polymerase chain reaction
RT-PCR: Reverse transcription PCR
qPCR: Quantitative real-time PCR
qRT-PCR: Quantitative real-time RT-PCR
RNAi: RNA interference
dsRNA: Double stranded RNA
siRNA: Small interfering RNA
RFP: Red fluorescent protein
EGFP: Enhanced green fluorescent protein
GFP: Green fluorescent protein
CRISPR: Clustered regularly interspaced short palindromic repeats
Cas9: CRISPR-associated protein 9
gRNA: Guide RNA
KO: Knock-out
WT: Wild type
AMPs: Antimicrobial peptides
A4: A4 promoter
