# Supplementary material for: Downregulation of BmSTAT transcription factor promoted nucleopolyhedrovirus replication in Bombyx mori
Source: Front Microbiol. 2024 Oct 23;15:1485951. doi: 10.3389/fmicb.2024.1485951 (PMC11537930; doi:10.3389/fmicb.2024.1485951)
Supplement: Supplementary file 1 [file Data_Sheet_1.docx]

# Supplementary Materials

## Table S1. The primers used in this study.

| Primers name | Sequences 5’-3’ |
| --- | --- |
| *BmSTAT-S* | F: GGATCCATGTACCCATACGATGTTCCAGATTACGCT  ACGCTGTGGACGCGAGCAC |
|  | R: GCGGCCGCTCAATGGTGATGGTGATGATGTATCAAGTCAATCATGTAC |
| *BmSTAT-L* | F: GGATCCATGTACCCATACGATGTTCCAGATTACGCT  ACGCTGTGGACGCGAGCAC |
|  | R: GCGGCCGCTCAATGGTGATGGTGATGATGCTTCAAGCACATGTCACTG |
| qPCR-*BmSTAT-S* | F: GAAAACGAAATGTTAAAGAA |
|  | R: ATCAAGTCAATCATGTACCT |
| qPCR-*BmSTAT-L* | F: CTGAAAACGAAATGTTAAAGAA |
|  | R: AACAAGTCGCTATTCATCAGGC |
| qPCR-*GAPDH* | F: CATTCCGCGTCCCTGTTGCTAAT |
|  | R: GCTGCCTCCTTGACCTTTTGC |
| qPCR-*GP41* | F: CGTAGTAGTAGTAATCGCCGC |
|  | R: AGTCGAGTCGCGTCGCTTT |
| qPCR-*BmSTAT* | F: GTCTTGGATCGAGAGCAGAATA |
|  | R: CATCTTCGTAACGAACATGTCC |
| ds*BmSTAT* | F: TAATACGACTCACTATAGGGACCGAGAGGAAGTACAGCGA |
|  | R: TAATACGACTCACTATAGGGCTCCTGGATCGTGTTGAGGT |
| dsRED | F: TAATACGACTCACTATAGGGGTACGGCTCCAAGGTGTACG |
|  | R: TAATACGACTCACTATAGGGGGTGTAGTCCTCGTTGTGGG |

## Table S2. siRNA sequence.

| Genes | Sequences 5’-3’ |
| --- | --- |
| *BmSTAT-S*，siRNA | Sense：GUACAUGAUUGACUUGAUAUGA |
|  | Antisense：UCAUAUCAAGUCAAUCAUGUAC |
| *BmSTAT-L*，siRNA | Sense：GCGACUUGUUCGAGCAGAUTT |
|  | Antisense：AUCUGCUCGAACAAGUCGCTT |

## Supplementary sequences

ds*BmSTAT*+A3intron: GGATCCCTCCTGGATCGTGTTGAGGTTGGACTGCATCGGGACCCCGTTGCCGCTCAGCTGTTGCTCCCTTTTCCATTTGATGAGCTCATCGTCCAACACCTGGCTCTGCAGCTGCCTCAGGTTCGTGATGTTCTCCTTCATGTGGTCCACCAGCTCCATTTGAGATTGAGTGATCTGCGCCACCAGCGCGTTGAGTTTCCTCTCCGTGTCCTCGATCTGTCCGCGCAGACAGGCCACCAGGTCGCGGCGCTCGGTGGTCACCGTCTGCTGCTGGAGGTAGTTCATGTGACCTTTGTTCTTCAAGCACTCGTGGTACTGCAGAGACAGGGATTCAATATTGGCCTGGAGGCTGCGGATTTCCTCGCCGGCTATGTTCACCTTTTGCCTTACAGTCTGCAGGCCCGTGATCAGCTCGCTGTACTTCCTCTCGGTGTGAGCTCATCGATTCTGGACTATGCACTTCGCCTCTCGGCCGGTGGGCCGTTATCGACCGTTATCTGACGAATGACTTTGTTCTGTTTCAGGAATTC

FXHBds*BmSTAT*:

GAATTCACCGAGAGGAAGTACAGCGAGCTGATCACGGGCCTGCAGACTGTAAGGCAAAAGGTGAACATAGCCGGCGAGGAAATCCGCAGCCTCCAGGCCAATATTGAATCCCTGTCTCTGCAGTACCACGAGTGCTTGAAGAACAAAGGTCACATGAACTACCTCCAGCAGCAGACGGTGACCACCGAGCGCCGCGACCTGGTGGCCTGTCTGCGCGGACAGATCGAGGACACGGAGAGGAAACTCAACGCGCTGGTGGCGCAGATCACTCAATCTCAAATGGAGCTGGTGGACCACATGAAGGAGAACATCACGAACCTGAGGCAGCTGCAGAGCCAGGTGTTGGACGATGAGCTCATCAAATGGAAAAGGGAGCAACAGCTGAGCGGCAACGGGGTCCCGATGCAGTCCAACCTCAACACGATCCAGGAGGCGGCCGC
